# COVID-19, stigma, and habituation: evidence from mobility data

**DOI:** 10.1186/s12889-023-14980-w

**Published:** 2023-01-13

**Authors:** Kenichi Kurita, Yuya Katafuchi, Shunsuke Managi

**Affiliations:** 1 Department of Environmental Changes, Faculty of Social and Cultural Studies, Kyushu University, Fukuoka, Japan; 2 Urban Institute, Kyushu University, Fukuoka, Japan; 3 Research Institute for Humanity and Nature, Kyoto, Japan; 4 Department of Cvilil Engineering, Faculty of Engineering, Kyushu University, Fukuoka, Japan

**Keywords:** COVID-19, Infection disease, Stigma, Self-restraint behavior, Non-pharmaceutical intervention, Mobility data

## Abstract

**Background:**

The Japanese government has restricted people’s going-out behavior by declaring a non-punitive state of emergency several times under COVID-19. This study aims to analyze how multiple policy interventions that impose non-legally binding restrictions on behavior associate with people’s going-out.

**Theory:**

This study models the stigma model of self-restraint behavior under the pandemic with habituation effects. The theoretical result indicates that the state of emergency’s self-restraint effects weaken with the number of times.

**Methods:**

The empirical analysis examines the impact of emergency declarations on going-out behavior using a prefecture-level daily panel dataset. The dataset includes Google’s going-out behavior data, the Japanese government’s policy interventions based on emergency declarations, and covariates that affect going-out behavior, such as weather and holidays.

**Results:**

First, for multiple emergency declarations from the beginning of the pandemic to 2021, the negative association between emergency declarations and mobility was confirmed in a model that did not distinguish the number of emergency declarations. Second, in the model that considers the number of declarations, the negative association was found to decrease with the number of declarations.

**Conclusion:**

These empirical analyses are consistent with the results of theoretical analyses, which show that the negative association between people’s going-out behavior and emergency declarations decreases in magnitude as the number of declarations increases.

## Background

The new coronavirus infection (COVID-19) has caused a global pandemic with about 217 million cases, and 4.5 million deaths as of 31 August 2021 [1]. Countries around the world that have anticipated or already suffered a catastrophic loss of life and economic damage from this pandemic have adopted a range of policies [2–4]. These policies have had a wide range of objectives, from saving the lives of those already infected to stopping the outbreak. The former policy interventions include subsidizing healthcare systems and preventing severe disease through rapid vaccination against COVID-19. On the other hand, the latter policy interventions have been designed to reduce opportunities for people to come into contact with COVID-19, as the majority of infections are airborne and droplet-transmitted [5].

Policies to reduce contact with these people have been implemented by restricting their behavior. Restrictions on people’s behavior have been adopted in various ways, including restricting gathering, restricting commuting to workplaces, and restricting going-out itself. For example, concerning the policy of restricting gatherings, Germany has notified that on 30 December 2020, private gatherings will be restricted to one household or another [6]; in terms of restrictions on commuting to workplaces, the state of Michigan in the US has issued a regulation on 12 November 2020 that imposes a fine of $7,000 on employers who are able to work remotely if they do not have an appropriate policy in place or a response plan in place [7]; restrictions on going-out were introduced in the UK on 4 January 2021, when the fine imposed on those who break a stay-home order was increased to £200  [8]. There are also differences between countries and local authorities regarding whether there are penalties, i.e., legally binding or not, for restricting people’s behavior.

As the policy mentioned above, interventions restricting people’s behavior illustrate that many countries prescribe penalties for these restrictions. On the other hand, some countries have adopted policies that rely on non-legally binding restrictions on behavior, i.e., voluntary action (i.e., self-restraint). For example, these non-legally binding policies have been implemented through requests to the public by state representatives or by declaring a state of emergency. Japan, which has adopted non-legally binding policies, has kept the number of infections and deaths under control compared to 36 other developed countries in the OECD, based on the government’s declaration of a state of emergency, which includes a call for individuals to refrain from going-out.

**Fig. 1:**
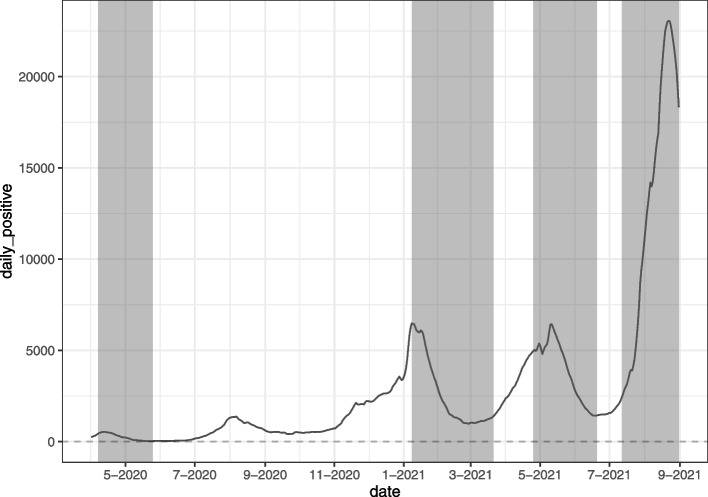
Trend of positive cases of COVID-19 and status of state of emergency of Japan. *Notes:* The solid line indicates 7-day moving average of daily COVID-19 positive cases in Japan. The shaded areas indicate the status of the declaration of a state of emergency in Tokyo prefecture, Japan, i.e., the date on which a state of emergency has been declared in Tokyo. The sample covers the period 1 April 2020 to 31 August 2021. *Source:* [11, 12] and authors’ calculation

**Fig. 2:**
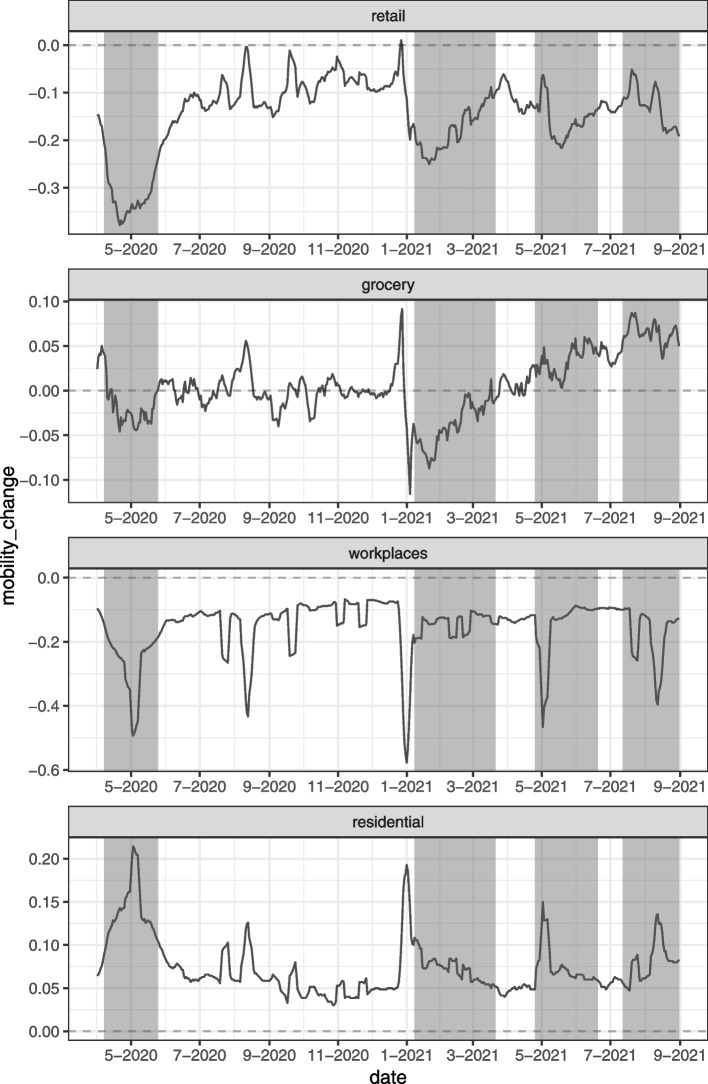
Mobility trend and status of the state of emergency of Japan. *Notes:* The solid lines represent the 7-day moving average of the change in the amount of movement across Japan for each category. The shaded areas indicate the status of the declaration of a state of emergency in Tokyo prefecture, Japan, i.e., the date on which a state of emergency has been declared in Tokyo. The category names at the top of each panel correspond to “Retail and recreation”, “Grocery and pharmacy”, “Workplaces”, and “Residential” from the top and indicate the amount of mobility change for each. The sample covers the period 1 April 2020 to 31 August 2021. *Source:* [11, 13] and authors’ calculation

The Japanese government, which has controlled the COVID-19 pandemic situation better than other industrialized countries, has restricted people’s behavior by declaring a state of emergency, despite having adopted a policy of no penalties and relying solely on people’s self-restraint called *Jishuku* in Japanese. The emergency declarations are designed to exercise authority and alert the public to the emergency and consist of requests to refrain from going-out unnecessarily, to refrain from holding public events, to refrain from opening restaurants, entertainment venues, and large mass merchandisers, and to shorten the opening hours of these facilities [9, 10]. Until now, the Government of Japan has issued these emergency declarations on a prefecture-by-prefecture basis, depending on the status of COVID-19 infection. Figure 1 shows the status of the emergency declarations in Tokyo and the COVID-19 infection status^1^. From the infection situation in Japan, we can confirm that the first wave of the COVID-19 epidemic started in April 2020, the second wave in August 2020, the third wave in December 2020, the fourth wave in April 2021, and the fifth wave in July 2021. On the other hand, from the emergency declarations issued for Tokyo, it can be confirmed that the first emergency declaration was issued from April to May 2020 during the first wave, the second from January 2021 to March 2021 during the third wave, the third from April 2021 to June 2021 during the fourth wave, and the fourth from July 2021 onward before the fifth wave. This figure highlights the fact that the government of Japan has declared a state of emergency to improve the situation of COVID-19 infection. On the other hand, in order to understand how the public has responded to the non-legally binding policy interventions through going-out activities, Fig. 2 shows the changes in the volume of going-out for the four categories “Retail and recreation”, “Grocery and pharmacy”, “Workplaces” and “Residential” retrieved from Google [13] and the declaration of a state of emergency in Tokyo prefecture. The figure can be summarized by the fact that the first emergency declarations show a significant decrease in going-out (and increase in time spent at home). In contrast, the second emergency declaration does not seem to have the same effect as the first and shows an increasing trend in mobility (and a decreasing trend in time spent at home). Furthermore, it can be confirmed that the amount of decrease in mobility (increase in the amount of time spent at home) under such emergency declarations tends to decrease with the number of times the emergency is declared.

The first emergency declaration issued by the Government of Japan in 2020, shown in the Figures mentioned above Figs. 1 and 2, is seen as having been successful in reducing the contact opportunities represented by people’s going-out behavior [14]. However, the second, third, and fourth emergency declarations, issued in 2021, have not yet been tested for effectiveness and have criticized [15–19].

Given this situation in Japan, the questions that this paper seeks to answer are as follows; first, what happens to people’s going-out behavior when they experience multiple declarations of a state of emergency, i.e., multiple policy interventions that impose non-legally binding restrictions on behavior? Second, in light of the first question, whether the second, third, and fourth declaration of a state of emergency reduced people’s going-out behavior. In the following, we review the studies related to these questions.

There is a wide range of literature on the analysis of mobility in relation to COVID-19 outside Japan [20–23]. For example, Delussu et al. (2022) [24] investigate a change in adherence to tiered restrictions in Italy. However, concerning the analysis of COVID-19-related mobility outside Japan, almost all studies have analyzed mobility in terms of legally binding policy interventions represented by punitive lockdowns.

On the other hand, various studies have been conducted on Japan’s self-restraint behavior [25–28]. Furthermore, there is a growing body of research on social stigma and social pressures related to COVID-19 [29–33]. However, there are no studies on self-restraint behavior considering habituation based on the number of emergency declarations.

Katafuchi et al. (2021) [14] and Kurita and Managi (2022) [28] are two existing studies on the analysis of stigma-focused Japanese non-legally binding policy interventions on going-out behavioral self-restraint, which is the scope of this study. Katafuchi et al. (2021) [14] discuss this relationship from theoretical and empirical analysis as in this study. However, the theoretical analysis in the paper does not incorporate the effect of voluntary restraint depending on the number of emergency declarations. The empirical analysis estimates the voluntary restraint effect of the policy intervention using a sample that includes only the first emergency declaration. Kurita and Managi (2022) [28] extend Katafuchi et al. (2021) [14] to analyze the dynamic model endogenizing an infection risk in a framework of evolutionary game. They conduct a welfare analysis showing that the emergency state increases social welfare. This study investigates the multiple emergency declarations not considered in the above studies.

Based on the background, the research question, and the review of previous papers on policy interventions for COVID-19 described above, the contribution of this paper is described as follows: First, this paper demonstrates habituation effects on self-restraint behavior under multiple non-legally binding policies. Specifically, this is achieved by presenting an economic theory model in which the number of announcements without penalty changes the effect of the announcements on going-out behavior. Second, this paper describes how the second and further announcements have been associated with people’s behavior concerning Japan’s non-legally binding policy, namely the declaration of a state of emergency. Specifically, we construct prefectural and daily panel data on going-out behavior and emergency declarations and covariates expected to affect going-out behavior and use the data to empirically show the impact of the second, third and fourth emergency declaration through estimations of econometric models.

The rest of the paper is organized as follows. First, in Theory, we use a theoretical model to analyze the impact of announcements on going-out behavior, considering that announcements are made multiple times. Second, in Methods, we construct a daily and prefectural panel dataset consisting of secondary data on emergency declarations, going-out behavior, and covariates. In Results, we then conduct an empirical analysis using this dataset. Finally, we conclude in Conclusion.

## Theory

We present a theoretical model of stigma following going-out behavior. The basic setting of the model follows Katafuchi et al. (2021) [14] and Kurita and Managi (2022) [28] while we extend it so that the effect varies with the number of emergency declarations (announcements), as described below.

Consider an economy where the population is normalized to 1. Individuals make decisions regarding two types of behavior: going-out or staying home. The payoff when choosing going-out is as follows:

1 $$\begin{aligned} u_\mathrm {out} - \phi [\gamma c + \iota \sigma e ^{-h(n)}s(x)]^{\delta }, \end{aligned}$$

the payoff when choosing staying home is as follows:

2 $$\begin{aligned} u_\mathrm {home}. \end{aligned}$$

Here, $u_\mathrm {out}$ and $u_\mathrm {home}$ are utility from going-out and that from staying home. The second term in (1) is the total psychological cost and the cost contains two factors: $\phi$ is the sensitivity of psychological costs, $F(\cdot )$ is the distribution function, $F'(\cdot ) = f(\cdot )$, the infection risk ($\gamma c$), social stigma ($\sigma s(x)$). $\gamma$ is the infection probability, $\delta$ is the cost to scale parameter, *c* is the cost, $\sigma$ is the relative impact of stigma, *s* is the stigma cost and $s'(\cdot ) < 0$. $\iota \in \{0, 1\}$ is the policy indicator variable, and $n = 1, 2, ...$ is the number of times that the state of emergency is implemented. $e^{-h(n)}$ represents the effect of stigma costs decreasing with the number of times that the state of emergency is implemented, and $h(\cdot )$ is an increasing function with *n*. This is inspired by *habituation* effect [34] and it is not taken into account by Katafuchi et al. (2021) [14] and Kurita and Managi (2022) [28].

We define the critical level of the sensitivity to psychological costs as follows:

3 $$\begin{aligned} u_\mathrm {out} - \hat{\phi }[\gamma c + \iota \sigma e ^{-h(n)}s(x)]^{\delta } = u_\mathrm {home}. \end{aligned}$$

From Eq. (3), players with sensitivities $\phi \le \hat{\phi }$ choose going-out meanwhile players with sensitivities $\phi > \hat{\phi }$. We get the following:

4 $$\begin{aligned} \hat{\phi } = \frac{u_\mathrm {out} - u_\mathrm {home}}{[\gamma c + \iota \sigma e ^{-h(n)}s]^{\delta }}. \end{aligned}$$

The population share of players who go out is given by

5 $$\begin{aligned} x = \Pr {(\phi \le \hat{\phi })} = F(\hat{\phi }). \end{aligned}$$

We assume that the stigma cost is an decreasing function with the share of players going-out, *x*, formally, $s = g(x)$, $g'(\cdot ) < 0$, $s \in [0, +\infty )$, and $s(1) > 0$.

The fixed point of the following equations corresponds to the equilibrium in this model:

6 $$\begin{aligned} \left\{ \begin{array}{cl} \hat{\phi } &{}= \frac{u_\mathrm {out} - u_\mathrm {home}}{[\gamma c + \iota \sigma e^{-h(n)}s]^{\delta }}, \\ x &{}= F \left( \hat{\phi }\right) , \\ s &{}= s(x). \end{array}\right. \end{aligned}$$

Summarizing Eq. (6), we define the function $\chi (x)$ as follows:

7 $$\begin{aligned} \chi (x) = F\left( \frac{u_\mathrm {out} - u_\mathrm {home}}{[\gamma c + \iota \sigma e ^{-h(n)}s(x)]^{\delta }}\right) , \end{aligned}$$

Therefore, the fixed point in $x = \chi (x)$, $x^{*}$, is the equilibrium population share of players who go out. To distinguish the population share of players who go out between under and without a state of emergency, we denote the former as $x_{1}$ and the latter as $x_{0}$.

### Proposition 1

Without the state of emergency, there exist an unique interior equilibrium as follows:

8 $$\begin{aligned} x_{0}^{*} = F\left( \frac{u_\mathrm {out} - u_\mathrm {home}}{(\gamma c)^{\delta }}\right) . \end{aligned}$$

Under the state of emergency, there can be multiple equilibria, $x_{1}^{*} \in \{x_{1,1}^{*},..., x_{1,k}^{*}\}$, $x_{1,1}^{*}< x_{1,2}^{*}<...< x_{1,k}^{*}$, *k* is positive integer greater than or equal to one.

### Proof

Proof is the same way as [14].

Proposition 1 shows same results as Katafuchi et al. (2021) [14]. Since we focus the effect of the number of times that the state of emergency is implemented on the self-restraint behavior, we do not discuss the multiplicity of equilibria. We define the self-restraint effect, *R*, as follows:

9 $$\begin{aligned} R := x_{0}^{*} - x_{1}^{*}. \end{aligned}$$

It means that the state of emergency has the self-restraint effect if $R > 0$.

### Proposition 2

The state of emergency has a self-restraint effect on going-out behavior.

### Proof

The maximum value of $\chi (x)|_{\iota = 1}$ is $\chi (1)|_{\iota = 1}$ because $\chi (x)|_{\iota = 1}$ is an increasing function with *x*. Comparing $\chi (1)|_{\iota = 1}$ with $x_{0}^{*}$, we obtain as follows:

10 $$\begin{aligned} \chi (1) - x_{0}^{*} = F\left( \frac{u_\mathrm {out} - u_\mathrm {home}}{[\gamma c + \sigma e ^{-h(n)}s(1)]^{\delta }}\right) - F\left( \frac{u_\mathrm {out} - u_\mathrm {home}}{(\gamma c)^{\delta }}\right) < 0, \end{aligned}$$

because

11 $$\begin{aligned}{}[\gamma c + \iota \sigma e ^{-h(n)}s(1)]^{\delta } > (\gamma c)^{\delta }. \end{aligned}$$

The equilibrium level of $x_{1}$ is less than $\chi (1)$. Therefore,

12 $$\begin{aligned} R > 0. \end{aligned}$$

The effect of the number of times that the state of emergency on the self-restraint behavior is summarized in the following proposition:

### Proposition 3

13 $$\begin{aligned} \frac{\partial R}{\partial n} < 0. \end{aligned}$$

### Proof

14 $$\begin{aligned} \frac{\partial R}{\partial n} = - \frac{\partial x_{1}^{*}}{\partial n}. \end{aligned}$$

Here,

15 $$\begin{aligned} \frac{\partial x_{1}^{*}}{\partial n} = \frac{\frac{\partial \chi (x_{1}^{*})}{\partial n}}{1 - \frac{\partial \chi (x_{1}^{*})}{\partial x}}. \end{aligned}$$

The denominator in Eq. (15) is positive by the following stability condition:

$$\begin{aligned} \frac{\partial \chi (x_{1}^{*})}{\partial x} < 1. \end{aligned}$$

Thus, the sign of Eq. (15) is positive because

$$\begin{aligned} \frac{\partial \chi (x_{1}^{*})}{\partial n} = f\left( \frac{u_\mathrm {out} - u_\mathrm {home}}{[\gamma c + \sigma e ^{-h(n)}s(x_{1}^{*})]^{\delta }}\right) (-\delta ) \left( \frac{u_\mathrm {out} - u_\mathrm {home}}{[\gamma c + \sigma e ^{-h(n)}s(x_{1}^{*})]^{\delta + 1}}\right) [-h'(n) \sigma e ^{-h(n)}s(x_{1}^{*})] > 0. \end{aligned}$$

Therefore, the sign of Eq. (14) is negative.

The implication of Proposition 3 is that the self-restraint effect of the state of emergency weakens with the number of times that the state of emergency is implemented. The result of Proposition 3 is consistent with an observation in Figs. 1 and 2. In the next section, we empirically test Proposition 3 using mobility data.

## Methods

### Econometric method

In order to identify how the first, second, third, and fourth emergency declarations issued by the Japanese government have been associated with people’s going-out behavior, this paper conducts an econometric analysis using secondary data. Specifically, we construct a panel data set including going-out behavior and some covariates that affect it, and try to estimate the association between mobility and emergency declaration by using the one-way error component model [35].

The model in the econometric analysis is as follows:

16 $$\begin{aligned} y_{it}= & {} \mathbf {x}'_{it} \varvec{\beta } + \varepsilon _{it}, \nonumber \\ \varepsilon _{it}= & {} \alpha _i + e_{it}, \end{aligned}$$

where *y* is the dependent variable of human flow, *i* is the index for the *i*th prefecture for $i = 1, \ldots , n$, *t* is the date for $t = 1, \ldots , T$, $\mathbf {x}$ is an explanatory variable vector containing covariates, $\varvec{\beta }$ is an unknown parameter vector, $\alpha$ is unobserved prefecture-level fixed-effect^2^ estimated through dummy variable for each prefecture *i*, $\varepsilon$ is the disturbance term, and *e* is stochastic variability. $\alpha$ is unobserved prefecture-level heterogeneity that is not dependent on time, such as prefecture-dependent idiosyncrasies related to the propensity to go out and industrial structure related to going-out behavior.

Due to its superiority in terms of interpretability, the model presented in Eq. (16) can be used as the main basis when analyzing the relationship between emergency declarations and going-outs. The issue is that this baseline model does not adequately account for autocorrelation, typically considered in spatiotemporal data structures. Therefore, in this study, the following spatial panel data model with temporal correlation [39, 40] is estimated in addition to Eq. (16):

17 $$\begin{aligned} y_{it}= & {} \mathbf {x}'_{it} \varvec{\beta } + \varepsilon _{it}, \\ \varvec{\varepsilon }_t= & {} \varvec{\alpha } + \varvec{\nu }_{t}, \nonumber \\ \varvec{\nu }_{t}= & {} \rho \mathbf {W} \varvec{\nu }_{t} + \varvec{\eta }_{t}, \nonumber \\ \varvec{\eta }_{t}= & {} \psi \varvec{\eta }_{t-1} + \mathbf {e}_{t}, \nonumber \end{aligned}$$

where $\varvec{\varepsilon }'_t = \left( \varepsilon _{1t}, \ldots , \varepsilon _{nt} \right)$ denotes regression disturbance, $\varvec{\alpha }' = \left( \alpha _1, \ldots , \alpha _n \right)$ is time-invariant region-specific random effects^3^, $\varvec{\nu }'_{t} = \left( \nu _{1t}, \ldots , \nu _{nt} \right)$ is spatial autocorrelation term, $\rho$ is spatial autocorrelation unknown coefficient, $\mathbf {W}$ is a $n \times n$ spatial weight matrix^4^, $\varvec{\eta }'_{t} = \left( \eta _{1t}, \ldots , \eta _{nt} \right)$ is temporal autocorrelation term, $\psi$ is temporal autocorrelation unknown coefficient, and $\mathbf {e}'_t = \left( e_{1t}, \ldots , e_{nt} \right)$ is stochastic variability.

### Data

The dependent variable used in this study is the Google COVID-19 Community Mobility Reports [13] as data showing the amount of change in people’s mobility. The dataset consists of the change in mobility against a reference value for six categories:^5^ retail and recreation, grocery and pharmacy, parks, transit stations, workplaces, and residential. Furthermore, the dataset consists of comprehensive data for Japan and data on changes in mobility at the sub-regional level, which comprises 47 prefectures.

This data is based on anonymized location data obtained from users of services using Google Account, including applications such as Google Maps, and from users using the Android operating system who have turned on the “Location History” setting. This data defines the number of visits as the volume of activity, except Residential^6^, and has the daily change in volume of activity relative to the median volume of activity for each day of the week between 3 January 2020 and 6 February 2020, before the spread of COVID-19.

In addition, to eliminate the trend by day of the week regarding the amount of mobility brought about by behavioral changes under COVID-19, such as the prevalence of work-from-home, as described in the introduction, we use a 7-day moving average for the mobility of the dependent variable. From the perspective of missing values, the mobility categories used in this analysis are the “Retail and recreation”, “Grocery and pharmacy”, “Workplaces”, and “Residential” categories in the Google COVID-19 Community Mobility Reports corresponding to the four dependent variables retail, grocery, workplaces, and residential, respectively. Furthermore, to confirm the robustness of this analysis, we also conduct an analysis using data obtained by Apple’s map application in addition to this data as a sensitivity analysis.

This paper defines the explanatory vector as:

$$\begin{aligned} \mathbf {x}_{it} := \left[ \mathbf {d}'_{it}, \mathbf {w}'_{it} \right] ', \end{aligned}$$

where $\mathbf {d}$ is vector of target variables, and $\mathbf {w}$ is covariate vector. The target explanatory variables in this paper are the emergency declarations issued by the Japanese government in 2020 and 2021. The date data on the emergency declarations are obtained from [11]. More specifically, we use a binary dummy variable as the target explanatory variable, which takes the value 1 when prefecture *i* is under a state of the emergency declaration at date *t*, and 0 otherwise.

The purpose of the empirical analysis in this study is to clarify the extent to which the change in mobility differs under the declaration of a state of emergency. As the first econometric model-based analysis in this study, the binary dummy variable $\texttt {emergency}_{it}$, which does not distinguish the number of emergency declarations, is used as the explanatory variable of interest in order to ascertain the pure correlation that exists between emergency declarations and the volume of mobility. As a second analysis using the econometric model, we use the binary dummy variables $\texttt {emergency\_1st}_{it}$, $\texttt {emergency\_2nd}_{it}$, $\texttt {emergency\_3rd}_{it}$, and $\texttt {emergency\_4th}_{it}$ as target explanatory variables in order to determine the extent to which people’s going-out behavior was associated with the policy intervention of declaring a state of emergency, depending on the number of times it was declared.

For the covariates vector, this paper includes weather information and prior information on the infection status of COVID-19 as factors that vary from prefecture to prefecture and from day to day and holiday information as factors that vary from day to day, which are likely to affect going-out behavior. We describe the detail of these covariates below.

First, weather data, consisting of daily precipitation $\texttt {precipitation}_{it}$, average temperature $\texttt {temperature}_{it}$, and average wind speed $\texttt {windspeed}_{it}$, obtained from the Japan Meteorological Agency is used as weather information^7^. The data observed at the prefectural capital of each prefecture is used as the weather information data for that prefecture. Weather data is used here because precipitation, temperature, or wind speed is a factor that can be predicted in advance by weather forecasting and thus may affect the decision-making process for going-out. In order to deal with anomalies of precipitation and wind speed caused by disasters such as torrential rains and typhoons, both data are logarithmically converted from values adjusted by the average of all prefectures during the sample period. In addition, we have normalized the average temperature data.

Second, as prior information on COVID-19 infection status, we use the data obtained from [12] on the number of daily COVID-19 positive cases by prefecture, which is one day lag of the seven-day moving average to remove the day-of-week trend^8^,^9^,^10^ ($\texttt {positive\_per1000}_{it}$). Furthermore, since the size of the population is reflected in the actual size of the number of positive cases, we use the number of positive cases per 1,000 people using the 2020 population projection data from the Ministry of Internal Affairs and Communications in order to control for the effect of population on the number of positive cases and to make the covariate more representative of the reality of the pandemic situation. We consider that this variable allows us to control the impact of the number of COVID-19 positives by prefecture, which is reported daily in the news, on people’s decisions to go out or stay home on the following day.

Third, as a factor that does not vary by prefecture but varies with time, this study uses a binary dummy variable for national holidays that takes the value of 1 if it is a national holiday and 0 otherwise, which may affect people’s going-out behavior ($\texttt {national\_holiday}_t$). In addition, we use as $\texttt {unofficial\_holiday}_t$ a dummy variable that takes a value of 1 for days that are not designated as “national holidays” by Japanese law but on which people tend to take holidays^11^, and 0 otherwise. We expect these variables, $\texttt {national\_holiday}_t$ and $\texttt {unofficial\_holiday}_t$ to control for the considerable variation in people’s going-out behavior during national holidays observed in the changes in mobility as seen in the introduction.

These dependent variables, explanatory variables of interest, and covariate data will be combined to construct a prefecture-specific daily panel data set. Regarding data availability, the sample period is from 1 April 2020 to 31 August 2021. The number of prefectures in the sample is $n = 47$, the number of days in the sample is $T = 518$, and the sample size, therefore, is $N = nT = 47 \times 518 = 24,346$.

## Results

In this section, we use the secondary data described above to analyze how these declarations are associated with people’s going-out behavior in the prefectures in Japan that experienced the first and second emergency declarations. First, we provide an overview of how emergency declarations, the explanatory variable of most interest to us, have been issued.

Table 1 shows the period over which emergency declarations related to COVID-19 were issued in the early stage of the pandemic in 2020. Moreover, Table 2 shows the period over which emergency declarations related to COVID-19 were issued in 2021. Finally, Table 3 shows the list of prefectures with multiple declarations of emergency from first to fourth. As these tables show, the declaration of a state of emergency in 2020 was issued to all prefectures, but with a difference between the start date and the lift date, while the declaration of a state of emergency in 2021 was issued to a limited number of prefectures, with a difference between the start date and the lift date. Using this heterogeneity of emergency declarations at the prefecture and date level, this study analyzes the association between emergency declarations and going-out behavior.

**Table 1:** Range of emergency statement in relation to COVID-19 declared in 2020 for prefectures of Japan

prefecture_en	emergency_start	emergency_end	times
Chiba	2020-04-07	2020-05-25	1
Fukuoka	2020-04-07	2020-05-14	1
Hyogo	2020-04-07	2020-05-21	1
Kanagawa	2020-04-07	2020-05-25	1
Osaka	2020-04-07	2020-05-21	1
Saitama	2020-04-07	2020-05-25	1
Tokyo	2020-04-07	2020-05-25	1
Aichi	2020-04-16	2020-05-14	1
Akita	2020-04-16	2020-05-14	1
Aomori	2020-04-16	2020-05-14	1
Ehime	2020-04-16	2020-05-14	1
Fukui	2020-04-16	2020-05-14	1
Fukushima	2020-04-16	2020-05-14	1
Gifu	2020-04-16	2020-05-14	1
Gunma	2020-04-16	2020-05-14	1
Hiroshima	2020-04-16	2020-05-14	1
Hokkaido	2020-04-16	2020-05-25	1
Ibaraki	2020-04-16	2020-05-14	1
Ishikawa	2020-04-16	2020-05-14	1
Iwate	2020-04-16	2020-05-14	1
Kagawa	2020-04-16	2020-05-14	1
Kagoshima	2020-04-16	2020-05-14	1
Kochi	2020-04-16	2020-05-14	1
Kumamoto	2020-04-16	2020-05-14	1
Kyoto	2020-04-16	2020-05-21	1
Mie	2020-04-16	2020-05-14	1
Miyagi	2020-04-16	2020-05-14	1
Miyazaki	2020-04-16	2020-05-14	1
Nagano	2020-04-16	2020-05-14	1
Nagasaki	2020-04-16	2020-05-14	1
Nara	2020-04-16	2020-05-14	1
Niigata	2020-04-16	2020-05-14	1
Oita	2020-04-16	2020-05-14	1
Okayama	2020-04-16	2020-05-14	1
Okinawa	2020-04-16	2020-05-14	1
Saga	2020-04-16	2020-05-14	1
Shiga	2020-04-16	2020-05-14	1
Shimane	2020-04-16	2020-05-14	1
Shizuoka	2020-04-16	2020-05-14	1
Tochigi	2020-04-16	2020-05-14	1
Tokushima	2020-04-16	2020-05-14	1
Tottori	2020-04-16	2020-05-14	1
Toyama	2020-04-16	2020-05-14	1
Wakayama	2020-04-16	2020-05-14	1
Yamagata	2020-04-16	2020-05-14	1
Yamaguchi	2020-04-16	2020-05-14	1
Yamanashi	2020-04-16	2020-05-14	1

*Notes:* emergency_start indicates the date on which a state of emergency was declared for the prefecture indicated in the row, and emergency_end indicates the date on which the state of emergency was lifted. *Source:* [11]

**Table 2:** Range of emergency statement in relation to COVID-19 declared in 2021 for prefectures of Japan

prefecture_en	emergency_start	emergency_end	times
Chiba	2021-01-08	2021-03-21	2
Kanagawa	2021-01-08	2021-03-21	2
Saitama	2021-01-08	2021-03-21	2
Tokyo	2021-01-08	2021-03-21	2
Aichi	2021-01-14	2021-02-28	2
Fukuoka	2021-01-14	2021-02-28	2
Gifu	2021-01-14	2021-02-28	2
Hyogo	2021-01-14	2021-02-28	2
Kyoto	2021-01-14	2021-02-28	2
Osaka	2021-01-14	2021-02-28	2
Tochigi	2021-01-14	2021-02-07	2
Hyogo	2021-04-25	2021-06-20	3
Kyoto	2021-04-25	2021-06-20	3
Osaka	2021-04-25	2021-06-20	3
Tokyo	2021-04-25	2021-06-20	3
Aichi	2021-05-12	2021-06-20	3
Fukuoka	2021-05-12	2021-06-20	3
Hiroshima	2021-05-16	2021-06-20	2
Hokkaido	2021-05-16	2021-06-20	2
Okayama	2021-05-16	2021-06-20	2
Okinawa	2021-05-23	2021-09-30	2
Tokyo	2021-07-12	2021-09-30	4
Chiba	2021-08-02	2021-09-30	3
Kanagawa	2021-08-02	2021-09-30	3
Osaka	2021-08-02	2021-09-30	4
Saitama	2021-08-02	2021-09-30	3
Fukuoka	2021-08-20	2021-09-30	4
Gunma	2021-08-20	2021-09-30	2
Hyogo	2021-08-20	2021-09-30	4
Ibaraki	2021-08-20	2021-09-30	2
Kyoto	2021-08-20	2021-09-30	4
Shizuoka	2021-08-20	2021-09-30	2
Tochigi	2021-08-20	2021-09-30	3
Aichi	2021-08-27	2021-09-30	4
Gifu	2021-08-27	2021-09-30	3
Hiroshima	2021-08-27	2021-09-30	3
Hokkaido	2021-08-27	2021-09-30	3
Mie	2021-08-27	2021-09-30	2
Miyagi	2021-08-27	2021-09-12	2
Okayama	2021-08-27	2021-09-12	3
Shiga	2021-08-27	2021-09-30	2

*Notes:* emergency_start indicates the date on which a state of emergency was declared for the prefecture indicated in the row, and emergency_end indicates the date on which the state of emergency was lifted. The missing value in emergency_end indicates that a state of emergency was in effect at the end of the sample period (31 August 2021). *Source:* [11]

**Table 3:** Prefectures of Japan experienced multiple emergency statements in relation to COVID-19

pref_1st	pref_2nd	pref_3rd	pref_4th
Aichi	Aichi	Aichi	Aichi
Akita	Chiba	Chiba	Fukuoka
Aomori	Fukuoka	Fukuoka	Hyogo
Chiba	Gifu	Gifu	Kyoto
Ehime	Gunma	Hiroshima	Osaka
Fukui	Hiroshima	Hokkaido	Tokyo
Fukuoka	Hokkaido	Hyogo	
Fukushima	Hyogo	Kanagawa	
Gifu	Ibaraki	Kyoto	
Gunma	Kanagawa	Okayama	
Hiroshima	Kyoto	Osaka	
Hokkaido	Mie	Saitama	
Hyogo	Miyagi	Tochigi	
Ibaraki	Okayama	Tokyo	
Ishikawa	Okinawa		
Iwate	Osaka		
Kagawa	Saitama		
Kagoshima	Shiga		
Kanagawa	Shizuoka		
Kochi	Tochigi		
Kumamoto	Tokyo		
Kyoto			
Mie			
Miyagi			
Miyazaki			
Nagano			
Nagasaki			
Nara			
Niigata			
Oita			
Okayama			
Okinawa			
Osaka			
Saga			
Saitama			
Shiga			
Shimane			
Shizuoka			
Tochigi			
Tokushima			
Tokyo			
Tottori			
Toyama			
Wakayama			
Yamagata			
Yamaguchi			
Yamanashi			

*Notes*: The prefectures included in each column indicate the first, second, third, and fourth emergency declarations issued

Before proceeding to the panel data model analysis, we first use descriptive statistical analysis to see how going-out behavior and COVID-19 infection status in Japan have changed over the sample period. Table 4 shows the monthly means of how the four explanatory variables of our panel data model, i.e., going-out, and one of the covariates, i.e., infection status, have changed across Japan. The table shows, first, that for the whole of Japan in the sample period, except grocery, mobility was lower than in the reference period [13] before the COVID-19 pandemic. Residential is positive in all periods, but since this is time spent at home, it can be interpreted as an increase in time spent at home, i.e., a decrease in going-out behavior, in all of Japan during the sample period compared to the reference period. Second, we can confirm that going-out behavior during the declaration of the state of emergency in 2020 (April and May 2020) and the initial declaration of the state of emergency in 2021 (January and February 2021) was reduced compared to before and after. Similar to the findings above, it is possible to identify a similar trend in residential. On the other hand, for the third and fourth emergency declarations after April 2021, it can be confirmed that it is difficult to interpret changes in the amount of mobility from this monthly average for all prefectures.

**Table 4:** Mean of mobility data and infection status by month for the whole Japan

year	month	retail	grocery	workplaces	residential	positive_per1000
2020	4	-0.293	-0.004	-0.216	0.120	0.0029
2020	5	-0.294	-0.020	-0.268	0.138	0.0010
2020	6	-0.139	0.002	-0.127	0.069	0.0004
2020	7	-0.114	-0.003	-0.145	0.070	0.0027
2020	8	-0.097	0.014	-0.196	0.075	0.0087
2020	9	-0.094	-0.010	-0.139	0.055	0.0044
2020	10	-0.079	-0.002	-0.092	0.041	0.0040
2020	11	-0.069	-0.002	-0.107	0.048	0.0098
2020	12	-0.075	0.011	-0.134	0.067	0.0193
2021	1	-0.211	-0.065	-0.201	0.098	0.0390
2021	2	-0.177	-0.031	-0.152	0.073	0.0167
2021	3	-0.109	-0.008	-0.124	0.052	0.0091
2021	4	-0.124	0.010	-0.145	0.056	0.0251
2021	5	-0.167	0.027	-0.181	0.083	0.0416
2021	6	-0.144	0.046	-0.096	0.059	0.0174
2021	7	-0.111	0.058	-0.135	0.063	0.0195
2021	8	-0.147	0.062	-0.201	0.092	0.1214

*Notes:* Each row shows the monthly level average for the whole Japan in the month indicated by the year-month pair. *Source:* [12, 13] and authors’ calculation

This study analyzes the association between the declaration of a state of emergency and going-out behavior using a panel data analysis rather than descriptive statistic analysis for the following reasons; first, it is difficult to establish precise treatment and control group for data in 2020. Second, it is also difficult to compare the association between mobility and the declaration of a state of emergency in 2020 with the association in 2021. Third, from the emergency declarations other than the initial one in 2021, it is impossible to correspond the setting of the treatment and control groups because the number of declarations is often different, even if they were issued during the same period in each prefecture.

Table 5 shows the correlation between the declaration of a state of emergency and going-out behavior under the control of daily variables influencing going-out behavior. In the table, the target explanatory variable for the emergency declaration is emergency, which is defined as a binary variable that does not distinguish the number of times of the declaration. The result, which is consistent with the empirical analysis conducted by Katafuchi et al. (2021) [14], suggests the possibility that the declaration of a state of emergency had a negative association with the going-out behavior (and that the declaration of a state of emergency had a positive association with the staying-home behavior) in terms of the comparative value of the going-out behavior with the pre-pandemic one.

**Table 5:** Result of panel data analysis for the association between emergency statement and mobility for the prefectures in Japan

dependent	explanatory	estimate	s.e.	p	covariates
retail	emergency	-0.1452	0.0046	<0.0001	Yes
grocery	emergency	-0.0236	0.0031	<0.0001	Yes
workplaces	emergency	-0.0764	0.0049	<0.0001	Yes
residential	emergency	0.0488	0.0022	<0.0001	Yes

*Notes:* The sample size is $N = nT = 47 \times 518 = 24,346$. The estimation results for each row show the coefficients estimated using the fixed-effect estimator. s.e. stands for cluster robust standard errors

Next, the results for the target explanatory variable, the declaration of emergency, distinguishing between the first (2020), second, third, and fourth (2021) declarations, i.e., emergency_1st, emergency_2nd, emergency_3rd, and emergency_4th, are shown in Table 6. The statistical significance of the estimated coefficients for the four target explanatory variables discussed above all show p-values below 5%, except for emergency_4th when retail is used as the dependent variable, emergency_3rd and emergency_4th for grocery, emergency_2nd and emergency_3rd for workplace, and emergency_4th for residential.

**Table 6:** Result of panel data analysis for the association between emergency statement and mobility for the prefectures in Japan, using divided emergency statement for 2020 and 2021

dependent	explanatory	estimate	s.e.	p	covariates
retail	emergency_1st	-0.2009	0.0064	<0.0001	Yes
	emergency_2nd	-0.0644	0.0057	<0.0001	
	emergency_3rd	-0.0605	0.0113	<0.0001	
	emergency_4th	-0.0088	0.0155	0.5692	
grocery	emergency_1st	-0.0314	0.0029	<0.0001	Yes
	emergency_2nd	-0.0195	0.0032	<0.0001	
	emergency_3rd	0.0080	0.0060	0.1814	
	emergency_4th	-0.0182	0.0110	0.0976	
workplaces	emergency_1st	-0.1316	0.0049	<0.0001	Yes
	emergency_2nd	0.0048	0.0030	0.1075	
	emergency_3rd	0.0063	0.0100	0.5263	
	emergency_4th	0.0428	0.0145	0.0030	
residential	emergency_1st	0.0765	0.0023	<0.0001	Yes
	emergency_2nd	0.0072	0.0011	<0.0001	
	emergency_3rd	0.0084	0.0041	0.0410	
	emergency_4th	-0.0129	0.0067	0.0522	

*Notes:* The sample size is $N = nT = 47 \times 518 = 24,346$. The estimation results for each row show the coefficients estimated using the fixed-effect estimator. s.e. stands for cluster robust standard errors

For mobility for retail, grocery, and workplace, we found statistically significant negative associations in declarations and mobility, except for emergency_4th in workplaces. On the other hand, in the model for residential, where the dependent variable is defined as the time at home, the exact opposite of the other dependent variables, a statistically significant positive association was found for declarations and time at home.

As for the magnitude of the coefficients, it can be confirmed that the coefficients become smaller as the number of emergency declarations increases in the model with the dependent variable of retail. Furthermore, in the models using workplace and residential, the coefficients increase with the number of emergency declarations (and decrease for residential).

The primary interpretation of the results is as follows: First, the estimation results of the model with retail as the dependent variable suggest that people refrained from going-out for retail and entertainment purposes during the first three emergency declarations. However, the degree of restraint may have decreased with each additional declaration. Second, the estimated coefficients for emergency declarations in the model with grocery as the dependent variable, i.e., the estimated coefficients for emergency declarations for going-out to purchase daily necessities such as food and medicine, indicate the possibility that the decrease in the restraint from going-out with an increase in the number of declarations, as seen in the results for retail, is unlikely to be reflected. Third, for workplaces, although people did not work in the workplace under the first declaration of emergency compared to before the pandemic, they may have worked in the workplace more in 2021, i.e., under the second and subsequent declarations of emergency, than before the pandemic. Fourth, the coefficient on emergency declarations in the model with residential as the dependent variable indicates that although the time spent at home increased under the first emergency declaration compared to the pre-pandemic period, the increase in time spent at home decreased in the second declaration. By the fourth declaration, the time spent at home may have decreased compared to the pre-pandemic period.

Even if a person has a sufficient subjective risk of COVID-19 infection and stigma against going-out, the purpose of going to a place represented by the grocery category may result in the need to go out to purchase items needed for survival. For going-out behavior for the purpose of going to a place represented by the workplaces category, people may also be required to commute by order of their company or their boss. Therefore, going-out for these purposes differs from retail and can be interpreted as going-out behavior that cannot be refrained from in some cases. In addition, since residential is a variable that indicates the time spent at home, it is subject to complex fluctuations depending on the types of going-out that can be restrained, such as retail, and the types of going-out that cannot be restrained, such as workplaces and grocery. Therefore, an increase in residential does not necessarily mean that people are refraining from going-out, and a decrease in residential does not necessarily mean that people are not refraining from going-out.

Taking into account these characteristics of categories of mobility, the results presented by Table 6 can be interpreted as follows: for *restraint-able* going-out (retail), voluntary restraint from going-out under a declared state of emergency has weakened as the number of the declarations increased, but this relationship could not be confirmed for *non-restraint-able* going-out (grocery and workplaces). Therefore, the empirical analysis of this study supports the results presented by the theoretical model, which shows that under a state of emergency people refrain from going-out, and that the negative association between mobility and state of emergency weakens with each successive declaration for those going-out that can be refrained from, where people have more freedom of decision-making.

In order to check the robustness of this relationship with an emergency and going-out behavior, the results of sensitivity analysis are presented below; first, the results without the addition of the covariate vector are presented in Tables 7 and 8. Second, the results for the different mobility datasets, COVID-19 Mobility Trends Reports [42] are shown in Tables 9 and 10. All of the sensitivity analysis generally supports the results presented in Tables 5 and 6.

**Table 7:** Result of panel data analysis for the association between emergency statement and mobility for the prefectures in Japan: sensitivity analysis without covariates

dependent	explanatory	estimate	s.e.	p	covariates
retail	emergency	-0.1452	0.0051	<0.0001	No
grocery	emergency	-0.0223	0.0033	<0.0001	No
workplaces	emergency	-0.0836	0.0060	<0.0001	No
residential	emergency	0.0510	0.0026	<0.0001	No

*Notes:* The sample size is $N = nT = 47 \times 518 = 24,346$. The estimation results for each row show the coefficients estimated using the fixed-effect estimator. s.e. stands for cluster robust standard errors

**Table 8:** Result of panel data analysis for the association between emergency statement and mobility for the prefectures in Japan, using divided emergency statement for 2020 and 2021: sensitivity analysis without covariates

dependent	explanatory	estimate	s.e.	p	covariates
retail	emergency_1st	-0.1961	0.0068	<0.0001	No
	emergency_2nd	-0.0861	0.0051	<0.0001	
	emergency_3rd	-0.0748	0.0084	<0.0001	
	emergency_4th	-0.0521	0.0069	<0.0001	
grocery	emergency_1st	-0.0376	0.0029	<0.0001	No
	emergency_2nd	-0.0266	0.0080	0.0009	
	emergency_3rd	0.0339	0.0053	<0.0001	
	emergency_4th	0.0514	0.0021	<0.0001	
workplaces	emergency_1st	-0.1409	0.0046	<0.0001	No
	emergency_2nd	-0.0042	0.0040	0.2947	
	emergency_3rd	-0.0181	0.0112	0.1068	
	emergency_4th	-0.0301	0.0133	0.0233	
residential	emergency_1st	0.0779	0.0023	<0.0001	No
	emergency_2nd	0.0141	0.0019	<0.0001	
	emergency_3rd	0.0201	0.0031	<0.0001	
	emergency_4th	0.0217	0.0022	<0.0001	

*Notes:* The sample size is $N = nT = 47 \times 518 = 24,346$. The estimation results for each row show the coefficients estimated using the fixed-effect estimator. s.e. stands for cluster robust standard errors

**Table 9:** Result of panel data analysis for the association between emergency statement and mobility for the prefectures in Japan: sensitivity analysis using Apple’s Data [42]

dependent	explanatory	estimate	s.e.	p	covariates
driving	emergency	-0.3803	0.0308	<0.0001	Yes
walking	emergency	-0.3759	0.0274	<0.0001	Yes

*Notes:* The sample size is $N = nT = 47 \times 518 = 24,346$. The estimation results for each row show the coefficients estimated using the fixed-effect estimator. s.e. stands for cluster robust standard errors

**Table 10:** Result of panel data analysis for the association between emergency statement and mobility for the prefectures in Japan, using divided emergency statement for 2020 and 2021: sensitivity analysis using Apple’s Data [42]

dependent	explanatory	estimate	s.e.	p	covariates
driving	emergency_1st	-0.5683	0.0136	<0.0001	Yes
	emergency_2nd	-0.0899	0.0191	<0.0001	
	emergency_3rd	-0.0837	0.0223	0.0002	
	emergency_4th	-0.1221	0.0435	0.0050	
walking	emergency_1st	-0.5233	0.0175	<0.0001	Yes
	emergency_2nd	-0.1596	0.0192	<0.0001	
	emergency_3rd	-0.1223	0.0280	<0.0001	
	emergency_4th	-0.1498	0.0423	0.0004	

*Notes:* The sample size is 47 prefectures between 1 April 2020 and 31 August 2021, i.e., $N = nT = 47 \times 518 = 24,346$. The estimation results for each row show the coefficients estimated using the fixed-effect estimator. s.e. stands for cluster robust standard errors

The findings of the baseline model based on Eq. (16) support the idea that the announcement of a state of emergency is inversely correlated with people’s propensity to leave their homes. Furthermore, the negative correlation between self-restraint-able going-out behavior and emergency declarations was found to be weaker with each announcement. However, the previous findings do not fully consider the autocorrelation present in the spatiotemporal nature of the panel data. In order to address this issue, Tables 11 and 12 present the estimation results of the model based on Eq. (17), which takes the spatiotemporal autocorrelation structure into account.

The results of Table 11 come from a model that considers the spatiotemporal structure but uses the same explanatory factors as those in Table 5, i.e., explanatory variables that do not distinguish between the number of emergency declarations. Like the previous results, all of the findings demonstrate that people tend to refrain from going-out behavior when non-legally binding policy interventions are in place. Additionally, the spatial and temporal autocorrelation coefficients ($\rho$ and $\psi$) are both positive and statistically significant, which distinguishes this model from the baseline model. These findings suggest that the relationship between emergency declarations and going-out behavior may be influenced by spatiotemporal patterns, and therefore warrants further investigation.

The results of Table 12, on the other hand, come from a model that takes into account the spatiotemporal structure and uses the same explanatory variables as Table 6, i.e., explanatory variables that distinguish the number of emergency declarations. This model also has positive and statistically significant spatial autocorrelation coefficients ($\rho$) and temporal autocorrelation coefficients ($\psi$), indicating that some consideration of spatiotemporal structure is important in examining the relationship between emergency declarations and going-out behavior. The coefficients for emergency declarations generally have similar signs and statistical significance to those in Table 6. One notable difference between the two tables (Tables 6 and 12) is the size of the coefficients for emergency declarations. Table 6 shows that for self-restraint-able going-out behavior (retail), the negative correlation between declaring a state of emergency and leaving home weakens as the number of times the state of emergency is declared increases. The results in Table 12, where retail is again the dependent variable, also display a roughly weakening trend. For instance, the second state of emergency declaration has a lesser negative association with going-out activity than the first. However, the third declaration’s negative correlation with going-out tends to be slightly stronger than the second one. This finding suggests that in a model that accounts for the spatiotemporal correlation structure, the negative correlation between emergency announcements and going-out behavior does not have a strong tendency to decline with each increase in the number of emergency declarations.

**Table 11:** Result of spatial panel data analysis for the association between emergency statement and mobility for the prefectures in Japan

dependent	explanatory	estimate	s.e.	p	covariates
retail	emergency	-0.0058	0.0006	<0.0001	Yes
	$\rho$	0.9951	0.0005	<0.0001	
	$\psi$	0.8739	0.0021	<0.0001	
grocery	emergency	-0.0016	0.0005	0.0015	Yes
	$\rho$	0.9900	0.0008	<0.0001	
	$\psi$	0.8438	0.0025	<0.0001	
workplaces	emergency	-0.0037	0.0004	<0.0001	Yes
	$\rho$	0.9943	0.0006	<0.0001	
	$\psi$	0.9630	0.0007	<0.0001	
residential	emergency	0.0017	0.0002	<0.0001	Yes
	$\rho$	0.9930	0.0007	<0.0001	
	$\psi$	0.9344	0.0011	<0.0001	

*Notes:* The sample size is $N = nT = 47 \times 518 = 24,346$. The estimation results for each row show the coefficients estimated using model (17). s.e. stands for cluster robust standard errors. The values corresponding to $\rho$ and $\psi$ represent the spatial and temporal autocorrelation coefficients, respectively

**Table 12:** Result of spatial panel data analysis for the association between emergency statement and mobility for the prefectures in Japan, using divided emergency statement for 2020 and 2021

dependent	explanatory	estimate	s.e.	p	covariates
retail	emergency_1st	-0.0078	0.0011	<0.0001	Yes
	emergency_2nd	-0.0051	0.0010	<0.0001	
	emergency_3rd	-0.0058	0.0013	<0.0001	
	emergency_4th	-0.0013	0.0021	0.5480	
	$\rho$	0.9951	0.0005	<0.0001	
	$\psi$	0.8740	0.0021	<0.0001	
grocery	emergency_1st	-0.0040	0.0009	<0.0001	Yes
	emergency_2nd	-0.0011	0.0008	0.1612	
	emergency_3rd	0.0001	0.0010	0.8886	
	emergency_4th	-0.0004	0.0017	0.8341	
	$\rho$	0.9900	0.0008	<0.0001	
	$\psi$	0.8440	0.0025	<0.0001	
workplaces	emergency_1st	-0.0057	0.0008	<0.0001	Yes
	emergency_2nd	-0.0038	0.0006	<0.0001	
	emergency_3rd	-0.0012	0.0008	0.1518	
	emergency_4th	-0.0039	0.0014	0.0048	
	$\rho$	0.9943	0.0006	<0.0001	
	$\psi$	0.9630	0.0007	<0.0001	
residential	emergency_1st	0.0025	0.0004	<0.0001	Yes
	emergency_2nd	0.0017	0.0003	<0.0001	
	emergency_3rd	0.0007	0.0004	0.0516	
	emergency_4th	0.0019	0.0006	0.0025	
	$\rho$	0.9930	0.0007	<0.0001	
	$\psi$	0.9344	0.0012	<0.0001	

*Notes:* The sample size is $N = nT = 47 \times 518 = 24,346$. The estimation results for each row show the coefficients estimated using model (17). s.e. stands for cluster robust standard errors. The values corresponding to $\rho$ and $\psi$ represent the spatial and temporal autocorrelation coefficients, respectively

In this section, we conducted an empirical analysis of the association between emergency declarations and voluntary restraint from going-out, considering the number of declarations. The results are consistent with the theoretical analysis conducted in Theory section, in that the declaration of a state of emergency has the effect of refraining from going-out, and that the effect of refraining from going-out decreases as the number of declarations of a state of emergency increases in the category of going-out that can be completely refrained from. The results of the empirical analysis were also shown to be robust by sensitivity analyses. In the model with spatiotemporal structure, the results were generally similar to those of the baseline model. However, the results also indicated a need for caution in terms of the magnitude of the coefficients.

## Conclusion

This study examined the effect of non-legally binding policy interventions on people’s going-out behavior when the number of interventions is increased from theoretical analysis and empirical analysis. In the theoretical analysis, we constructed a model that extends Katafuchi et al. (2021) [14] so that the effect of the policy intervention changes with the number of times the announcement is executed. Furthermore, in comparative statics using the model, we confirmed that the effect of the policy intervention on the suppression of going-out declines with each increase in the number of implementations. In the empirical analysis, we developed a daily panel dataset at the prefectural level, focusing on the declaration of a state of emergency, a non-legally binding policy intervention in Japan to change behavior to mitigate the disadvantages arising from COVID-19. Furthermore, using the data, we analyzed the relationship between the four emergency declarations and going-out behavior and found that the negative association between emergency and mobility weakened as the number of emergency declarations increased in the analysis of going-out behavior related to the objective category with a high degree of freedom to refrain from going-out, which is consistent with the theoretical model. The results of the model with the spatiotemporal structure were generally similar to those of the baseline model. However, the results indicated that the magnitude of the coefficients should be interpreted with caution.

In light of the findings of this study, namely that similar emergency declarations have a diminishing effect on behavior change with each successive declaration, we suggest that policymakers should make more fundamental changes to the requests and punitive nature of emergency declarations to make them more progressive and practical in their policy interventions.

In this study, there are several limitations. First, this study has not been able to identify exact causal effects. To resolve this issue, we would like to conduct future analyses using experimental data. Second, the generalizability of the results of this study to further policy interventions should be carefully considered, given that only the association in question has been clarified. In other words, the results of this study’s analysis cannot be said to have external validity. We believe that this problem can also be resolved by clarifying the causal effects of multiple emergency declarations on going-out behavior, either by using experimental data or by using a natural experimental situation. Third, of the data used in the analysis of this study, the sample size for the fourth declaration of a state of emergency is relatively small (see Table 3). Therefore, the results regarding the association between the fourth declaration of a state of emergency and going-out behavior are not statistically significant in most models, and caution should be exercised in interpreting these results. However, it is important to note that, as the results of this study show, the association between the first through the third declarations of a state of emergency and self-restraint from going-out follows our hypothesis to a certain extent.

The change in going-out behavior due to the declaration of emergency is heterogeneous across occupations and industries^12^. This may be due to whether remote work is possible or not. However, even if remote work is possible and the types of jobs are similar, the changes in going-out behavior may differ across firms. One hypothesis is that the stigma of not coming to work (i.e., the stigma of working remotely) may change depending on how often one’s colleagues come to work. We will analyze this hypothesis by constructing a social norm model for each workplace for future work.

## Data Availability

This study uses the publicly available datasets to analyze how policy interventions aimed at reducing the spread of COVID-19 with respect to Japanese prefectures have associated with going-out behavior. Data on going-out behavior for the four categories used as outcomes in this statistical analysis can be downloaded from the Google Community Mobility Report (https://www.google.com/covid19/mobility/). Data on the implementation of prefectural emergency declarations in Japan, the focus of this study, are available at the github repository “covid-19_emergency_statement_japan” (https://github.com/yuya-katafuchi/covid-19_emergency_statement_japan). In addition, this study has put in place prefecture- and time-dependent observable confounding factors in the statistical analysis: (1) weather data (precipitation, wind speed, and temperature) at the prefectural level is available for download from the Japan Meteorological Agency (https://www.data.jma.go.jp/gmd/risk/obsdl/index.php), (2) the number of COVID-19 cases at the prefectural level is available for download from the TOYO KEIZAI ONLINE (https://toyokeizai.net/sp/visual/tko/covid19/), and (3) dates of the official holidays designated by Japanese government can be downloaded at Cabinet Office of Government of Japan (https://www8.cao.go.jp/chosei/shukujitsu/gaiyou.html). The alternative data of going-out behavior in our sensitivity analyses is available at Apple Mobility Trend Reports (https://covid19.apple.com/mobility).

## Footnotes

1 In Figures 1 and 2, the reason for focusing on Tokyo as the target area for the declaration of a state of emergency can be summarized in three points: First, the duration of the declaration of a state of emergency is different for each prefecture. The second reason is that Tokyo is the most populous prefecture in Japan. Third, because the number of emergency declarations issued in Tokyo and their total duration are the highest and longest among the prefectures in Japan.

2 We estimate the model including the fixed-effect by the least squares method. The reason is two points; first, this study focuses on the association between emergency declarations and mobility and does not focus on the association between time-independent characteristics and mobility across prefectures[36, 37]. In addition, it did not focus on differences in the association between time-independent properties and mobility across prefectures and individuals[36]. Second, the simplicity of the model and estimation[37, 38].

3 The use of random effect estimation in this study is motivated by its ability to encompass the fixed effect estimator with spatial autocorrelation [41], as well as for ease of computation. This allows for a more comprehensive analysis of the data while also making the estimation process more efficient.

4 In this study, the 4-nearest neighbor method is used to produce the proximity matrix for calculating the spatial weight matrix. Importantly, some prefectures in our data, such as Hokkaido and Okinawa, do not share borders with other prefectures. Therefore, the matrix is not based on the queen and rook criteria, which are commonly used to generate proximity matrices.

5 “Retail and recreation” refers to visits to entertainment venues, including restaurants, shopping centers, museums, and other shopping and experiential facilities; “Grocery and pharmacy” refers to visits to grocery shops, drugstores, and other facilities where people can purchase daily essentials; “Parks” refers to visits to parks, including national parks and gardens; “Transit stations” refers to visits to public transport hubs; “Workplaces” refers to visits to workplaces; “Residential” refers to targeted time spent at home. For more detail on mobility data, see [13].

6 For Residential, the time spent is counted as the amount of mobility, not the number of visits.

7 Data on sunlight are similarly available, but were not included as explanatory variables in the models estimated in this paper because of their complete multicollinearity with precipitation.

8 In Japan, there is a trend in the number of positive cases by day of the week, with a significant decrease in the number of positive cases the day after the weekend and a national holiday. It has been suggested that this trend may be a manifestation of the strategy of health authorities and hospitals with COVID-19 testing resources to deliberately reduce the number of tests on weekends and national holidays in order to prevent pressure on medical conditions such as the number of hospital beds.

9 The model in this paper incorporates one day lag of seven-days moving average of COVID-19 positive cases. However, people may make decisions concerning the number of COVID-19-positive cases from a few days earlier. Following this argument, we estimated the model by replacing the one-day lag of the seven-day moving average of COVID-19 cases with two, three, four, five, six, or seven days lag. However, the results did not change (data not shown).

10 There is seasonality in going-out behavior. For example, it is possible that it is more difficult to refrain from going-out in the summer than in the winter. This seasonality is, however, also controlled by covariates related to weather.

11 In this paper, we define new year holidays and Obon holidays as unofficial_holiday for which this variable takes the value 1. Specifically, we define the new year holidays as January 2 and 3 in 2021 and the Obon holidays as 13 August to 16 in 2020 and 2021 as unofficial_holiday.

12 For example, in a survey in Japan, about 50% of consultants are able to telework, but less than about 5% of drivers are able to telework [43].

## References

[1] World Health Organization. WHO Coronavirus (COVID-19) Dashboard. 2021. https://covid19.who.int/. Accessed 31 Aug 2021.

[2] Martin A, Markhvida M, Hallegatte S, Walsh B. Socio-Economic Impacts of COVID-19 on Household Consumption and Poverty. Econ Disasters Clim Change. 2020;4:453–79. 10.1007/s41885-020-00070-3. 10.1007/s41885-020-00070-3 PMC7376321 32838120

[3] Mandel A, Veetil V. The Economic Cost of COVID Lockdowns: An Out-of-Equilibrium Analysis. Econ Disasters Clim Change. 2020;4:431–51. 10.1007/s41885-020-00066-z. 10.1007/s41885-020-00066-z PMC7304379 32838118

[4] Gharehgozli O, Nayebvali P, Gharehgozli A, Zamanian Z. Impact of COVID-19 on the Economic Output of the US Outbreak’s Epicenter. Econ Disasters Clim Change. 2020;4:561–73. 10.1007/s41885-020-00069-w. 10.1007/s41885-020-00069-w PMC7372201 32838119

[5] Bahl P, Doolan C, de Silva C, Chughtai AA, Bourouiba L, MacIntyre CR (2020). Airborne or Droplet Precautions for Health Workers Treating Coronavirus Disease 2019?. J Infect Dis..

[6] deutschland de. The Federal Government informs about the corona crisis. 2020. https://www.deutschland.de/en/news/german-federal-government-informs-about-the-corona-crisis. Accessed 1 Apr 2021.

[7] State of Michigan. As COVID-19 cases rise, State emphasizes worker protections in offices, remote work policies. 2020. https://www.michigan.gov/coronavirus/0,9753,7-406-98158-544922--,00.html. Accessed 1 Apr 2021.

[8] nidirect. Coronavirus (COVID-19) regulations: compliance and penalties. 2020. https://www.nidirect.gov.uk/articles/coronavirus-covid-19-regulations-compliance-and-penalties. Accessed 1 Apr 2021.

[9] Cabinet Secretariat, Japan. Revision of the Basic Policy on Countermeasures against Novel Coronavirus Disease (in Japanese). 2021. https://corona.go.jp/emergency/pdf/kihonhoushin_kaitei_20210202.pdf. Accessed 1 Apr 2021.

[10] Ministry of Health, Labour, and Welfare, Japan. Overview of the Act on Special Measures for Pandemic Influenza and New Infectious Diseases Preparedness and Response. 2020. https://www.mhlw.go.jp/content/10900000/000606693.pdf. Accessed 1 Apr 2021.

[11] Katafuchi Y. covid-19_emergency_statement_japan. 2020. https://github.com/yuya-katafuchi/covid-19_emergency_statement_japan. Accessed 11 July 2020.

[12] TOYO KEIZAI ONLINE. Coronavirus Disease (COVID-19) Situation Report in Japan. 2020. https://github.com/kaz-ogiwara/covid19/blob/master/README.en.md. Accessed 11 July 2020.

[13] Google. Google COVID-19 Community Mobility Reports. 2021. https://www.google.com/covid19/mobility/. Accessed 1 Apr 2021.

[14] Katafuchi Y, Kurita K, Managi S (2021). COVID-19 with stigma: Theory and evidence from mobility data. Econ Disasters Clim Change..

[15] The Mainichi. Tokyo-area train commuter figures dropped only slightly under 2nd virus state of emergency. 2021. https://www3.nhk.or.jp/news/special/coronavirus/emergency_2021/detail/detail_16.html. Accessed 1 Apr 2021.

[16] Jiji Press. Coronavirus Remains Vigorous in 2 Weeks of Emergency in Japan, Infecting 85,000. 2021. https://sp.m.jiji.com/english/show/9910. Accessed 1 Apr 2021.

[17] At Press. The first questionnaire survey on state of emergency (in Japanese). 2021. https://www.atpress.ne.jp/news/251171. Accessed 1 Apr 2021.

[18] The Yomiuri Shimbun. Weekend travel on the rise a month into state of emergency. 2021. https://the-japan-news.com/news/article/0007130899. Accessed 1 Apr 2021.

[19] Reuters. Japan extends COVID emergency in Tokyo, PM Suga says Olympics still going ahead. 2021. 2021;33(4):1–23. https://www.reuters.com/world/asia-pacific/japan-government-seeks-extend-state-emergency-may-31-2021-05-07/. Accessed 5 June 2021.

[20] Basellini U, Alburez-Gutierrez D, Fava ED, Perrotta D, Bonetti M, Camarda CG (2021). Linking excess mortality to mobility data during the first wave of COVID-19 in England and Wales. SSM Popul Health..

[21] Devaraj S, Patel PC (2021). Change in psychological distress in response to changes in reduced mobility during the early 2020 COVID-19 pandemic: Evidence of modest effects from the US. Soc Sci Med..

[22] Velias A, Georganas S, Vandoros S (2022). COVID-19: Early evening curfews and mobility. Soc Sci Med..

[23] Carroll R, Prentice CR (2021). Community vulnerability and mobility: What matters most in spatio-temporal modeling of the COVID-19 pandemic?. Soc Sci Med..

[24] Delussu F, Tizzoni M, Gauvin L (2022). Evidence of pandemic fatigue associated with stricter tiered COVID-19 restrictions. PLOS Digit Health..

[25] Hanibuchi T, Yabe N, Nakaya T (2021). Who is staying home and who is not? Demographic, socioeconomic, and geographic differences in time spent outside the home during the COVID-19 outbreak in Japan. Prev Med Rep..

[26] Katafuchi Y. Residential land price fluctuations caused by behavioral changes on work-from-home based on COVID-19. MPRA Paper. 2021;1–24. https://mpra.ub.uni-muenchen.de/110091/. Accessed 1 Oct 2022.

[27] Katsuki R, Kubo H, Yamakawa I, Shinfuku N, Sartorius N, Sakamoto S, et al. Association between Self-Restraint Behavior, Stigma and Depressive Tendency in Office Workers during the COVID-19 Pandemic in Japan–Self-Restraint Behavior and Depression during the COVID-19. Psychiatry Int. 2021;2(3):300–9.

[28] Kurita K, Managi S. COVID-19 and stigma: Evolution of self-restraint behavior. Dyn Game Appl. 2022;1–15. 10.1007/s13235-022-00426-2 PMC8788405 35096465

[29] Badrfam R, Zandifar A. Stigma over COVID-19; new conception beyond individual sense. Arch Med Res. 2020;51(6):593–4. 10.1016/j.arcmed.2020.05.006 PMC7237948 32467051

[30] Bagcchi S (2020). Stigma during the COVID-19 pandemic. Lancet Infect Dis..

[31] Jecker NS, Takahashi S. Shaming and Stigmatizing Healthcare Workers in Japan During the COVID-19 Pandemic. Public Health Ethics. 2021;14(1):72–78.

[32] Takahashi R, Tanaka K. Social punishment for breaching restrictions during the COVID-19 pandemic. Econ Inq. 2021;59(4):1467–82. 10.1111/ecin.13020. 10.1111/ecin.13020 PMC8444821 34548708

[33] Wright J. Overcoming political distrust: the role of ‘self-restraint’ in Japan’s public health response to COVID-19. Japan Forum. 2021;33(4):1–23.

[34] Dodge R (1923). Habituation to Rotation. J Exp Psychol..

[35] Baltagi BH (1984). A Monte Carlo Study for Pooling Time Series of Cross-Section Data in the Simultaneous Equations Model. Int Econ Rev..

[36] Gelman A. Analysis of Variance: Why It Is More Important than Ever. Ann Stat. 2005;33(1):1–31. http://www.jstor.org/stable/3448650.

[37] Bell A, Jones K (2014). Explaining Fixed Effects: Random Effects Modeling of Time-Series Cross-Sectional and Panel Data. Political Sci Res Methods..

[38] Pinker R. "Stigma and social welfare." Social Work (1939-1970). 1970;27(4):13-17.

[39] Baltagi BH, Song SH, Jung BC, Koh W (2007). Testing for serial correlation, spatial autocorrelation and random effects using panel data. J Econ..

[40] Millo G (2014). Maximum likelihood estimation of spatially and serially correlated panels with random effects. Comput Stat Data Anal..

[41] Elhorst JP (2003). Specification and estimation of spatial panel data models. Int Reg Sci Rev..

[42] Apple. Apple COVID-19 Mobility Trends Reports. 2021. https://covid19.apple.com/mobility. Accessed 1 Apr 2021.

[43] Kawaguchi D, Motegi H (2021). Who can work from home? The roles of job tasks and HRM practices. J Jpn Int Econ..

